# A randomized controlled trial investigation of a non-stimulant in attention deficit hyperactivity disorder (ACTION): Rationale and design

**DOI:** 10.1186/1745-6215-12-77

**Published:** 2011-03-13

**Authors:** Tracey W Tsang, Michael R Kohn, Daniel F Hermens, Simon D Clarke, C Richard Clark, Daryl Efron, Noel Cranswick, Chris Lamb, Leanne M Williams

**Affiliations:** 1 Brain Dynamics Centre, Sydney Medical School and Westmead Millennium Institute, Sydney, New South Wales, Australia; 2 Sydney Medical School, University of Sydney, Sydney, New South Wales, Australia; 3 Centre for Research into Adolescents' Health, Department of Adolescent Medicine, Westmead Hospital and The Children's Hospital at Westmead, Westmead, New South Wales, Australia; 4 Cognitive Neuroscience Laboratory, School of Psychology, Flinders University, Adelaide, South Australia, Australia; 5 Brain Health Clinics, Adelaide, South Australia, Australia; 6 Department of General Paediatrics, Royal Children's Hospital, Parkville, Melbourne, Victoria, Australia; 7 Clinical Pharmacology and Australian Paediatric Pharmacology Research Unit, Murdoch Children's Research Institute and Royal Children's Hospital, Parkville, Melbourne, Victoria, Australia; 8 Child Development Unit, Women's and Children's Hospital, North Adelaide, South Australia, Australia; 9 Clinical Research Unit, Brain and Mind Research Institute, University of Sydney, Sydney, Australia; 10 Previous Address: Brain Dynamics Centre, Sydney Medical School and Westmead Millennium Institute, Sydney, New South Wales, Australia

## Abstract

**Background:**

The ACTION study (*Attention deficit hyperactivity disorder Controlled Trial Investigation Of a Non-stimulant) *is a multi-center, double-blind, randomized cross-over trial of the non-stimulant medication, Atomoxetine, in children and adolescents with attention deficit hyperactivity disorder (ADHD). The primary aims are to examine the efficacy of atomoxetine for improving cognition and emotional function in ADHD and whether any improvements in these outcomes are more pronounced in participants with comorbid anxiety; and to determine if changes in these outcomes after atomoxetine are more reliable than changes in diagnostic symptoms of ADHD. This manuscript will describe the methodology and rationale for the ACTION study.

**Methods:**

Children and adolescents aged 6 - 17 y with ADHD will be enrolled. Clinical interview and validated scales will be used to confirm diagnosis and screen for exclusion criteria, which include concurrent stimulant use, and comorbid psychiatric or neurological conditions other than anxiety. Three assessment sessions will be conducted over the 13-week study period: Session 1 (Baseline, pre-treatment), Session 2 (six weeks, atomoxetine or placebo), and Session 3 (13 weeks, cross-over after one-week washout period). The standardized touch-screen battery, "IntegNeuro™", will be used to assess cognitive and emotional function. The primary measure of response will be symptom ratings, while quality of life will be a secondary outcome. Logistic regression will be used to determine predictors of treatment response, while repeated measures of analysis will determine any differences in effect of atomoxetine and placebo.

**Results:**

The methodology for the ACTION study has been detailed.

**Conclusions:**

The ACTION study is the first controlled trial to investigate the efficacy of atomoxetine using objective cognitive and emotional function markers, and whether these objective measures predict outcomes with atomoxetine in ADHD with and without comorbid anxiety. First enrollment was in March 2008. The outcomes of this study will be a significant step towards a 'personalized medicine' (and therefore a more efficient) approach to ADHD treatment.

**Trial registration:**

Australian and New Zealand Clinical Trials Registry ANZCTRN12607000535471.

## Background

Attention deficit hyperactivity disorder (ADHD) affects at least one child or young person in every classroom worldwide, with prevalence estimates ranging between 2 and 16% [1]. It is the most common psychiatric disorder in children and adolescents, and continues into adulthood in a majority of cases [2]. Problems experienced by children and adolescents with ADHD include difficulties with sustaining attention and/or hyperactivity/impulsivity. These problems impact unfavorably on the young person's everyday functioning [3], as well as the health-related quality of life of themselves and their families [4].

Stimulant medications are the most common pharmacologic treatment for ADHD [5], acutely improving symptoms in 60 - 90% of patients [2,6-8]. However, when stimulant drugs are not effective, or when there are contraindications to stimulants, non-stimulant drugs are a viable alternative. Increasingly, non-stimulant medications such as atomoxetine (ATMX) are considered when comorbid conditions, such as anxiety, are present [9]. ATMX is purported to be a viable alternative to stimulant treatment for ADHD, with particular efficacy for children and adolescents with ADHD who are prone to problems with inhibition, anxiety and substance abuse [10].

ATMX has been approved by the US Food and Drug Administration for the treatment of ADHD [7], and has a demonstrated response rate of up to 63.5% [11-13], comparable to that of stimulant treatment [14]. Compared to stimulant drugs, ATMX has a lower potential for abuse or misuse [7,15], and has the advantage of once daily dosing and continuous coverage [15]. To date, the focus has been on efficacy studies, to determine if treatment with ATMX produces improvement in diagnostic ADHD symptoms. Symptoms are assessed using well-established rating scales, including the ADHD Rating Scale IV - Parent version (ADHD-RS IV) score [3,11,16-28], Clinical Global Impressions (CGI) score [17,29], and Connors' Parent and Teacher Rating Scales: Short version [13], and the Parent-rated Hyperactivity/Impulsivity Swanson Nolan and Pelham ratings [29]. In terms of the effect of ADHD medications on objective cognitive outcomes, one cross-sectional study using historical data indicated that young people who were medicated for ADHD (with either ATMX, methylphenidate or amphetamine) performed superiorly on objective cognitive tests compared to their non-medicated ADHD counterparts, although their performance remained poorer than that of non-ADHD controls [30]. To our knowledge, there have been no controlled trials undertaken to determine: 1) if ATMX has efficacy for improving cognition; and 2) if response to ATMX is predicted by other measures of cognition in ADHD, with and without comorbid anxiety. The ACTION study has been designed to address this issue.

The ACTION study will be a double-blind, randomized, controlled cross-over trial, to assess ADHD at baseline, and after treatment with either ATMX or placebo. At each assessment point clinical ratings of symptoms will be acquired. The ACTION study will assess cognition using a standardized, computerized battery called 'IntegNeuro™" (Brain Resource Ltd., Sydney, Australia and San Francisco, USA). IntegNeuro™ has been validated and established in ADHD [1,31,32], thereby providing a basis for future studies seeking to extend or replicate our findings. Cognitive markers from IntegNeuro™ have been found to identify ADHD with high sensitivity and specificity [1]. These markers also correlate with brain function assessed by both EEG and heart rate variability [1].

The findings from the ACTION study will help support a more personalized approach to treatment options available for children and adolescents with ADHD by examining the efficacy of ATMX in improving both subjectively-rated symptom measures and objectively-assessed performance in ADHD. It will also provide evidence about which objective cognitive markers relate to ATMX response in ADHD, and how anxiety moderates ATMX response. An evidence-base of this kind may help support clinicians in making decisions about if and when to consider non-stimulants as an alternative to stimulants or non-pharmacologic treatments in each individual child or adolescent.

The primary aims of the ACTION study are to:

1. Assess whether ATMX has efficacy in improving cognition and emotional function in children and adolescents with ADHD.

2. Assess whether presence of co-morbid anxiety influences the response to ATMX in children and adolescents with ADHD in relation to cognition and emotional function.

3. Ascertain the reliability of cognitive and emotional function changes after ATMX compared to clinical symptoms of ADHD, and how these correlate with symptom changes. Please refer to Section 5 for more detail.

The methodology for the ACTION study will be detailed in this manuscript.

## Methods/Design

### 1. Study regimens

**Figure 1:**
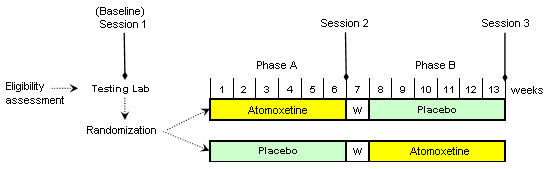
**Illustration of the ACTION study schema**. W = washout period. Eligibility assessment (screening) included the ADHD-RS IV and ADISC. Sessions 1, 2, and 3 included ADHD-RS IV, CPRS-R, DASS, BRISC, CGI, PedsQL (parent and teenager reports), and the IntegNeuro™battery. The WPREMB-R was to be done on a weekly basis during Phases A and B.

**Figure 2:**
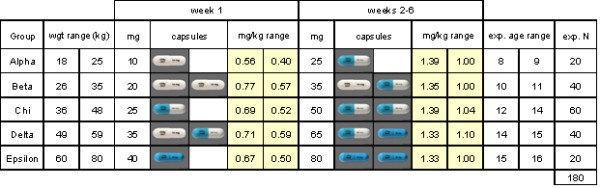
**Atomoxetine dosage schedule**. Week 1 is the initiation dose, with approximately 0.5 mg.kg^-1 ^for each patient group; weeks 2-6 reach the target dose of approximately 1.2 mg.kg^-1 ^for each group (with minimum dose of 1.0 mg.kg^-1 ^and maximum dose of 1.4 mg.kg^-1^).

Eligible and consenting participants will complete rating scales and a computer-based test battery to assess cognitive performance. Figure 1 shows the schema for the ACTION study, which is a randomized double-blind cross-over study. After baseline assessment, participants will be randomized to receive either ATMX (brand name: Strattera) or placebo (starch powder with silicone 5% in a gelatin capsule) first, for six weeks (Phase A), before undergoing a one-week "washout" period (no capsules). Cross-over will occur after this washout period, and participants will receive the other compound (i.e., the compound they did not receive during Phase A) over a second six-week period (Phase B). Baseline (Session 1) assessments will be repeated at all time-points. ATMX doses will be based on the participant's weight, according to five pre-determined weight groups, covering the predicted weight ranges from 18 to 80 kg (Figure 2). Both the ATMX and the placebo capsules will look identical, and were purchased from Eli Lilly Australia.

Randomization will be performed by a hospital biostatistician using a centralized, pre-determined pair-wise randomization technique, ensuring an even allocation of participants to receiving ATMX or placebo first; while also taking into account the expected ratios across the five weight groups (Figure 2). Once the randomization list has been generated, the hospital biostatistician will forward a copy of the master list to the compounding chemist responsible for packaging and labeling the blister cards for the study. The investigators, referring clinicians, and the participants will remain blinded to group allocation. All capsule packages will be prepared prior to the commencement of the trial, and packages will be dispensed according to the pre-determined randomization master list. As each participant enters the study, they will be allocated a study ID code in the order of numerical sequence. The corresponding blister cards will be labeled with the ID codes and corresponding study phase only. The participating clinicians, investigators, and the participants will be blinded to group allocation until the completion of the study.

#### 1.1. Treatment delivery

The dose schedule for ATMX will be based on recommendations from clinical practice and previous literature (Figure 2) [18,33]. In addition to the drug blister packs, a personalized "Take Home Package" booklet will be provided to parents/guardians of the participants, which will include a Day Counter log book to help parents keep track of their child's compliance (via daily tick boxes parents can mark off whenever their child takes their daily dose). Participants will be asked to take their capsules at the same time each morning. Drug blister packs and "Take Home Package" booklets for use during Phase A and B will be given to the parents at the end of Session 1 and Session 2 respectively. At the subsequent appointment, parents will be required to return their completed "Take Home Package" booklet, along with the drug blister packs regardless of their child's compliance, to enable the investigators to record any missed doses.

#### 1.2. Study participants

**Figure 3:**
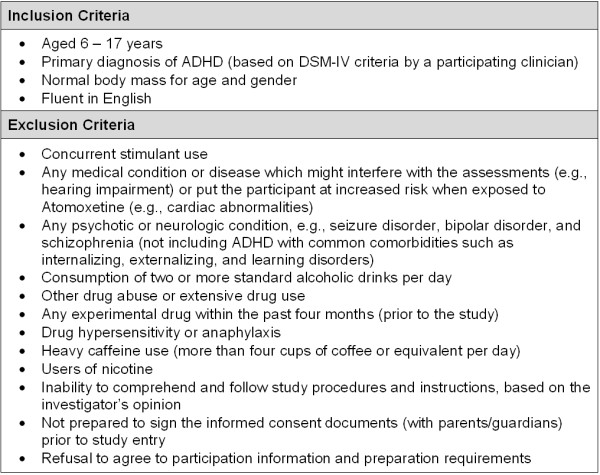
**Inclusion and Exclusion criteria for the ACTION study**. ADHD = attention deficit hyperactivity disorder; DSM-IV = Diagnosis and Statistical Manual of Mental Disorders, Edition 4.

Figure 3 lists the inclusion and exclusion criteria for the ACTION study. Eligibility criteria include normal body mass for age and gender [34], age of 6 to 17 years (inclusive), and a primary diagnosis of ADHD. Both boys and girls will be enrolled into the study. The presence of any of the exclusion criteria listed in Figure 3 will preclude the participant from enrolling into ACTION.

### 2. Participant study visits

#### 2.1. Screening (pre-Baseline)

Potentially eligible patients will undergo a clinical examination by a participating clinician. A primary diagnosis of ADHD will be based on *Diagnostic and Statistical Manual of Mental Disorders - Fourth edition *criteria [35]. The ADHD-RS IV will be used to confirm ADHD diagnosis, ADHD subtype, and to assess symptom severity. Additionally, the Anxiety Disorders Interview Schedule for Children (ADISC) [36] will be used to assess the presence of comorbid conditions. The ADHD-RS IV and ADISC scales are detailed in Section 4. They have been shown to have excellent reliability [36], validity [37], and medium-to-high specificity and sensitivity [38] in children and adolescents. The purpose of the Screening visit will be to confirm a primary diagnosis of ADHD, to determine symptom ratings, and eligibility for the study, as determined by participating clinicians.

Participants deemed eligible for the ACTION study will undergo a two-week washout period prior to Baseline testing (Session 1) if they are taking any ADHD medication at the time of Screening. Subsequent visits occur at weeks six and 13. Appointments will be confirmed over the telephone at least one day prior.

#### 2.2. Session 1 (Baseline; 0 weeks)

Prior to attending assessment sessions, participants will be asked to refrain from consuming caffeine that day (including coffee, chocolate milk, caffeinated soft-drinks).

Assessments will include cognitive and emotional function tasks using IntegNeuro™, body mass, and validated parent-reported scales for ADHD symptom severity and self-report questionnaires (Section 4). On average, assessments will take the participants approximately two hours to complete. Upon completion of Session 1, parents will be given their child's first course of study capsules and a "Take Home Package" booklet as mentioned above in 1.1.

#### 2.3. Session 2 (6 weeks; post-Phase A)

The assessments undertaken at Baseline (Session 1) will be repeated at Session 2. Parents will be reminded to return their child's capsule blister packs and "Take Home Package" booklet, so that the investigators can record compliance to the study drugs during each study phase. As with Session 1, on completion of Session 2 parents will be provided with their child's next course of study capsules and a second "Take Home Package" booklet for use during Phase B of the study.

#### 2.4. Session 3 (13 weeks; post-Phase B)

The assessments undertaken at Session 2 will be repeated at Session 3, with the exclusion of the provision of capsule blister packs and "Take Home Package" booklets.

#### 2.5. Ethical considerations

The ACTION study will be conducted according to the principles of the Declaration of Helsinki 2008, and International Conference on Harmonization (ICH) guidelines. Investigators ensure that "Good Clinical Practice" principles will be adhered to, as outlined in 21 FDA Code of Federal Regulations CFR 312, subpart D, "Responsibilities of Sponsors and Investigators," 21 CFR, part 50, 1998, and 21 CFR, part 56, 1998.

Institutional Review Board (IRB) approval is obtained prior to patient enrolment at any clinical site. All protocol modifications will be submitted to each IRB for approval before implementation. Prior to undertaking any study-related procedures, investigators will obtain written informed consent from each study participant after verbal and written explanation of study aims, methods, and potential hazards and benefits.

### 3. Study organization

#### 3.1. Organizational structure

#### The ACTION study will be conducted across three Australian sites

• Sydney: Brain Dynamics Centre laboratory located at Westmead Hospital (lead site). It is a center of the University of Sydney Medical School and has a partnership with the Centre for Research into Adolescents' Health (Department of Adolescent Medicine at the Children's Hospital at Westmead (CHW) and Westmead Hospital).

• Adelaide: Cognitive Neuroscience Laboratory located at School of Psychology, Flinders University. It has a partnership with a large community adolescent mental health practice in Adelaide.

• Melbourne: Department of General Paediatrics at the Royal Children's Hospital, Melbourne.

Clinical Trial Coordinators (CTCs) will be appointed at all three sites.

#### 3.2. Site selection/training/recruitment

Clinical sites were selected based on the likelihood of meeting recruitment goals and executing the protocol. During a site initiation visit, CTCs at each site will be trained in protocol implementation and data collection methods. CTCs will work closely with participants and clinicians, ensure that all instruments are completed by participants, and function as study coordinators (i.e., liaise among sites, data management at their own site, and ensure the smooth operations of the study at their site).

#### 3.3. Enrolment/randomization

Patients are referred to the ACTION study by participating clinicians, by faxing completed Clinical Package forms (containing basic demographic details, body mass; and completed ADHD-RS IV and ADISC) to their local CTC. The site's CTC will contact the interested patient's parent to formally invite them to the study, and provide additional information and consent forms.

### 4. Data collection

#### 4.1. Screening measures

Attention Deficit Hyperactivity Disorder Rating Scale-IV (ADHD-RS IV) [39] The ADHD-RS IV will confirm DSM-IV diagnosis and subtype of ADHD, indicated by a score >1 in six or more items in the Inattentive and/or Hyperactive-Impulsive sections of the scale. If a DSM-IV diagnosis cannot be confirmed using the ADHD-RS IV, the participant will be excluded. Total scores will be used as a measure of symptom severity.

Clinicians will also undertake the Anxiety Disorders Interview Schedule for DSM-IV: Child Version (ADISC: Parent Interview Schedule) [36]. Scores of ≥4 for any of the domains are indicative of a positive diagnosis. Participants will be categorized as having either ADHD + comorbid anxiety (i.e., Separation anxiety disorder, Obsessive compulsive disorder, and/or Generalized anxiety disorder [36]); or ADHD without anxiety (which includes those with other comorbidities excluding anxiety). The number of diagnosed comorbidities in total, including anxiety and others, will also be recorded.

#### 4.2. Baseline (Session 1), Session 2 and Session 3 measures

##### 4.2.1. ADHD symptom severity

At each testing session parents will complete the ADHD-RS IV, as a measure of ADHD symptom severity.

Parents will also rate symptom severity on the Conners' Parent Rating Scale - Revised (Long version; CPRS-R) [40]. The CPRS-R ratings will provide a second assessment of symptom severity, providing a within-study check on the consistency of symptom ratings. T-scores for all 13 subscales will be recorded, where larger T-scores indicate greater impairment.

The secondary outcomes of ACTION will be the relationships between the primary cognitive measures and ratings obtained from the ADHD-RS IV and CPRS-R.

##### 4.2.2. Self-reported negative feelings and self regulation

Negative feelings of anxiety, depression and stress will also be assessed via self-report, using previously established scales. The Depression Anxiety and Stress Scale (DASS) [41] has been established in 6 to 18 year olds with ADHD [42], and a child-worded version of this scale has been validated against the original in the same children completing both original (with parental help as needed to understand questions) and child version, in a randomized within-subjects design [43]. The DASS questionnaire will be scored for three domains: Depression, Anxiety, and Stress.

To assess state as well as trait anxiety, the State-Trait Anxiety Inventory (STAI) [44] or STAI for Children (STAIC) [45] will be used. The STAIC is suitable for use in children aged 6 to 14 years, while the STAI is suitable for those aged >14 years. The questionnaires provide scores on both State anxiety and Trait anxiety [46] and will be used at all three laboratory-based sessions.

To complement the scales assessing negative feelings, an established measure of self-regulation (the BRISC) will be used, which captures risk for brain health versus resilience and capacity for seeking social support [47]. The BRISC has been normed in 6 to 92 year olds [47], and there is good correspondence between child and adult-worded versions [41]. The BRISC score of Negativity Bias identifies a spectrum of brain health disorders (particularly anxiety and depression) with 87% sensitivity; and it is inversely related to Emotional resilience and social skills capacity (data not yet published). The validation study also established the convergence between this scale and a child-worded version in the same randomized within-subjects design [43]. The three BRISC scores are Negativity bias, Emotional resilience and Social skills.

#### 4.3. Other symptom measures

To further explore the secondary aims of the study, other measures of ADHD symptoms will be included at each assessment session.

##### Clinical Global Impression (CGI) scale [48]

The CGI Severity scale will be used at Session 1, based on the opinion of the investigator; while the CGI Improvement scale will be used at both Session 2 and Session 3, taking into account the opinions of both the investigator and the parent. Rating scores for each time-point will be recorded.

##### Pediatric Quality of Life Inventory (PedsQL) - Parent report, Version 4.0 [49]

The PedsQL - Parent reports for young children (8 to 12 years old) and adolescents (13 to 18 years old) were utilized at all three time-points to assess the health-related quality of life, based on the opinion of the parent. The two summary (mean) scores of Physical health (Physical health items) and Psychosocial health (Emotional, Social, and School functioning items) will be recorded.

##### PedsQL - Child report and PedsQL - Teenager report [49]

The PedsQL - Child report will be given to participants aged 8 to 12 years, while the PedsQL - Teenager report will be provided to those aged 13 to 18 years of age. Similarly to the PedsQL - Parent report, the summary scores for Physical health and Psychosocial health will be recorded for analysis.

##### Weekly Parent Rating of Evening and Morning Behavior - Revised (WPREMB-R)

This questionnaire was developed by Eli Lilly Pty. Ltd. for monitoring morning and late afternoon/evening behavior in children and adolescents with ADHD who are being treated using ATMX [50]. Six copies of the WPREMB-R will be included in the "Take Home Package 1" booklet (Phase A), and seven will be included in the "Take Home Package 2" booklet (Phase B), so that parents can complete the questionnaire at the start of each week during these phases of the study. The maximum possible score obtainable is 33, which is indicative of poorer ADHD behavior. Mean WPREMB-R scores will be calculated and recorded for both Phase A and Phase B. Missing responses in any week will exclude the participant's data for that study phase, since the scoring method of this questionnaire does not permit adjustment for missing information.

#### 4.4. IntegNeuro™battery

The IntegNeuro™ cognitive test battery is made up of 13 different tasks which require a total of approximately 50 minutes to complete. The 13 tasks which make up the IntegNeuro™ test battery were designed and validated to challenge participants in six different cognitive domains: i) Sensori-motor; ii) Learning and memory; iii) Language; iv) Attention and working memory; v) Executive function/planning; and vi) Emotion identification [1,31,32]. Table 1 lists the specific markers which will be investigated in ACTION for cognitive and emotional function and the tasks and measures used in IntegNeuro™ to ascertain them. Each marker is a composite of up to three tasks, and have a combined sensitivity of 88% in children and adolescents with ADHD [1]. The decision to focus on the Inhibition and Emotion identification markers as primary outcomes for the ACTION study was due to recent investigations [42,51], which led us to hypothesize that these markers would be more likely to improve after non-stimulant treatment in patients with ADHD and comorbid anxiety. Research into the cognitive markers in ADHD and their response to different ADHD treatments is still in the early stages so it would be premature to limit our investigations to only two of six markers which have been validated in ADHD. Hence, all six markers will be assessed in ACTION due to the novelty of this study. Marker scores are the focal dependent measures of interest, calculated as standardized scores for the tasks each marker comprises (Table 1). More detailed scores for each individual task will also be available for secondary analyses. Williams et al. (2010) provides a clear description of the tasks which will be used in ACTION [1].

**Table 1:** Six cognitive and emotional function markers, and their contributing measures

Marker	Summary definition	Task	Measure
Cognitive:			

1. Sustained attention (vigilance)	To maintain attention over time during continuous and repetitive activities.	Continuous performance task	Reaction time, total errors

2. Impulsivity	To initiate a behavior without adequate forethought; the inability to suppress automatic responses when they are inappropriate.	Continuous performance task Go-NoGo	Errors of commission Errors of commission

3. Intrusions	The repetition of erroneous responses, even in the absence of interfering stimuli.	Maze Switching of attention Verbal memory recall	Overrun errors Errors (digits and letters) Total intrusion errors

4. Inhibition	The inability to suppress task-irrelevant information.	Verbal interference	Errors (word), errors (color), errors (interference)

5. Response variability	The consistency of response time.	Continuous performance task Go-NoGo	Variability of reaction time Variability of reaction time

Emotional function:			

6. Emotion identification	The capacity to identify the facial expressions of basic emotion displayed by others.	Emotion identification task	Percentage correct, response time for each expression (fear, anger, sadness, happiness, disgust, neutral)

See Williams et al. (2010) for more detail about the tasks [1].

In the IntegNeuro™ cognitive battery, participants will hear the test instructions through a pair of headphones, and respond to the tasks by either speaking into the microphone (attached to the headphones) or using the touch-screen for non-verbal tasks. Participants will be supervised throughout the test via CCTV (closed-circuit television) from an adjacent room. Additional instructions will be given during the test if necessary. Participants will provide consent to be monitored via CCTV by signing a Surveillance Authority Form. The CCTV will only be used for the purpose of monitoring the patient during the visit and no recordings will be made.

To minimize the effect of familiarization and practice effects at subsequent testing sessions, IntegNeuro™ has a set of parallel forms for use in repeat testing sessions, whereby the sequences/words/patterns used in each of the tasks have been programmed to be different between the time-points. IntegNeuro™ will be set up to have the participant complete the test battery corresponding to their ACTION time-point.

### 5. Research endpoints

The primary endpoint will be clinically significant improvement in cognition and emotional function after ATMX treatment assessed using IntegNeuro™ (Table 1); and in ADHD symptoms (ADHD-RS IV, CGI, WPREMB, and CPRS-R subscales: Cognitive problems/inattention, Hyperactivity, Conners' ADHD index, CGI restless-impulsive, CGI total, DSM-IV inattentive, DSM-IV hyperactive-impulsive, and DSM-IV total). Further analyses will be undertaken to compare the differences in ATMX treatment effects between participants with and without comorbid anxiety; and between ADHD subtypes.

Part of the novelty of this trial lies in our utilization of objective assessments for cognition and emotional function. As such, we will also determine if our objective measures predict response to ATMX in ADHD, and in patients with comorbid anxiety. Treatment response is defined in Section 7.3.2.

The secondary endpoints will be improvements after ATMX treatment in quality of life (PedsQL - self and parent); emotional states and self-regulation (DASS-BRISC); and state and trait anxiety (CPRS-R subscales: Anxious-shy, STAI and STAIC). Previous ADHD treatment with stimulant medication and comorbid anxiety will be included as covariates.

### 6. Adverse events and safety monitoring

Parents are advised to contact the CTC in the case of any adverse events - defined as any physical, behavioral/psychological, or physiological problems potentially related to the study treatment or testing sessions. In the case of mild adverse reactions, the parent will be asked to continue on the study treatment and monitor the condition, informing the CTC if the problem persists or worsens. The study clinician will also be available for the CTC to obtain a sound medical opinion if required. If the adverse reaction is deemed an emergency, the parents will be advised to take the participant to the Emergency department of their nearest hospital. In these situations, the participant's group allocation will be revealed in order to inform the hospital of what the participant was administered. All adverse events will be recorded.

The investigators will be responsible for monitoring the safety of study participants and to take appropriate action concerning any event that seems unusual. They will ensure that appropriate medical care is maintained throughout the study and after the study to follow adverse events.

### 7. Statistical analysis

#### 7.1. Sample size

The data used for the power calculation of the inhibition function primary outcome measures are from a sample of 175 ADHD individuals assessed off and then on stimulant medication (methylphenidate or dexamphetamine; data not published). The power calculation of the emotional function primary outcome measures are based on a sample of 30 ADHD participants from the same sample.

Power was set at 0.9, alpha level at 0.05, and the mean difference (change in off-versus on-medication) and standard deviations for the mean of the n = 175 group was used. The means for the n = 30 group were set at '0', since there was no change expected in these outcome measures for the placebo group. After applying the pilot data means from each primary outcome measure the highest sample size estimate we obtained was n = 152 for each group under two-tailed conditions. Therefore, a minimum total of 152 participants are required in this cross-over study to achieve the desired level of probability in detecting a statistically significant difference across the two primary outcome measures.

#### 7.2. Data upload and reports

A computerized protocol will quantify cognitive data in a standardized manner. Responses obtained from the questionnaires will be paper-based, and entered into computer-based scoring spreadsheets which have been set up to automatically score each questionnaire. Raw and composite IntegNeuro™ data for all sites will be extracted and stored by the Brain Resource Company Ltd., which will be periodically forwarded to the Sydney site (once per month).

IntegNeuro™reports (from Brain Resource Company Ltd.) will be generated and sent to the referring study clinicians after Session 1 and Session 3 testing. These reports will present performance at Session 1 compared to normative data, and after Session 3 (comparing performance after Session 2 and Session 3, including comparisons to normative data).

#### 7.3. Data analysis

The ACTION study will adopt an all available data analytical approach, meaning that all data obtained will be included in analyses, regardless of adherence to the study. Accordingly, there will be no imputation of missing data. For all analyses, a *p *value of <0.05 will be considered statistically significant.

##### 7.3.1. Baseline analyses

Continuous data at baseline will be checked for normality of distribution and log-transformed if required. Logistic regression models will be used to determine whether or not the cognitive and emotion measures (using the composite scores) predict the presence of comorbid anxiety (as determined using the ADISC questionnaire).

##### 7.3.2. Predictors and moderators of response to treatment

Simple linear regression models will be used to observe any relationships between cognition/emotion composite scores at baseline and questionnaire scores after ATMX treatment to see if the objectively-measured cognition/emotion measures are related to the subjectively-measured ADHD symptom measures (ADHD-RS IV, CPRS-R). To examine the *predictive *relationship of cognition/emotion measures on ADHD symptom measures, changes after ATMX treatment in ADHD symptom measures will be categorized depending on whether or not they responded to the treatment. Analyses will be performed using two different definitions of treatment response: 1) ≥25% improvement and 2) ≥40% improvement; while non-response will be defined as 3) <25% improvement and 4) <40% improvement [11,17,19,52]. More specifically regarding the primary outcome, six predictors (the composite scores from baseline for the six cognition and emotional function markers, Table 1) will be used in logistic regression models to examine if baseline performance in these measures predict response to ATMX. Response in terms of the primary outcome will be defined initially as a ≥25% improvement (versus <25% improvement) in ADHD-RS IV and CPRS-R t scores (see Section 5). A secondary, more stringent definition of response will subsequently be implemented, using a response cut-point of ≥40%. With a planned sample size of >60, these analyses will be sufficiently powered. Further exploratory analyses may be performed at a later date.

Absolute change scores (continuous ADHD symptom data) will be used to see if the cognition/emotion measures *moderate *response to ATMX in ADHD symptom measures. These investigations will initially be performed on the total ADHD cohort, before examining any differences between those with and without comorbid anxiety.

##### 7.3.3. Post treatment

Repeated measures analysis of variance (ANOVA) will be performed on continuous variables (see Section 5), comparing the results obtained between baseline and ATMX, baseline and Placebo, and between ATMX and Placebo. Cohen's d effect size will be calculated using the mean differences between baseline and ATMX, and baseline and Placebo. Analyses will be performed in the total cohort, and then comparing those with and without comorbid anxiety. Presence/absence of previous stimulant treatment for ADHD, and an interaction term between presence/absence of comorbid anxiety and previous stimulant treatment will be used as covariates.

## Results and Discussion

The ACTION study will be a multi-site, double-blinded, randomized cross-over study examining the effects of ATMX on cognitive and emotional functions in children and adolescents with ADHD, whilst also identifying objective predictors of ATMX response in this group, including those with and without comorbid anxiety. The cross-over design of the study enables participants to act as their own controls when testing whether cognitive and emotional function at baseline is able to predict changes in symptom ratings after medication treatment. Enrolment and testing commenced in March 2008. Potential limitations in the study design pertain to the one-week washout period between treatment phases, and the absence of an "anxiety only" arm for examining comorbid anxiety outcomes. Although our selection of a washout period of one-week's duration was based on previous research [53-55], it is not known if any carry over effects were present. However, it is highly unlikely that the results obtained after six weeks of placebo treatment could be attributed to ATMX in those participants receiving active treatment (ATMX) in Phase A. Future studies may consider observing changes at first response and full response, and to vary the length of their washout periods between treatments. Additionally, an anxiety only arm should also be incorporated if investigating changes in comorbid anxiety outcomes.

## Conclusions

The detailed methodology for the ACTION study has been presented. The novelty of this trial lies in its utilization of objective measures for cognition and emotion; and in its combination of objective and subjective, commonly-used assessment tools to determine the efficacy of ATMX in ADHD with and without comorbid anxiety. This research will further our knowledge on the effective use of ATMX in improving symptoms in child and adolescent ADHD; providing additional evidence to aid in personalized treatment approaches.

## Abbreviations

ACTION: ADHD Controlled Trial Investigation Of a Non-stimulant; ADISC: Anxiety Disorders Interview Schedule for DSM-IV: Child version (Parent interview schedule); ADHD: Attention Deficit Hyperactivity Disorder; ADHD-RS IV: Attention Deficit Hyperactivity Disorder - Rating Scale IV; ANOVA: Analysis Of Variance; ATMX: Atomoxetine; BRISC: Brain Resource Inventory of Social Cognition; CCTV: Closed Circuit Television; CFR: Code of Federal Regulations; CGI: Clinical global impression scale; CHW: Children's Hospital at Westmead; CPRS-R: Conners' parent rating scale - revised (long version); CTCs: Clinical trial coordinators; DASS: Depression; anxiety and stress scale; DSM-IV: Diagnostic and Statistical Manual of Mental Disorders - Fourth edition; FDA: U.S. Food and Drug Administration; ICH: International Conference on Harmonization; ID: Identification; PedsQL: Pediatric quality of life inventory; STAI: State-trait anxiety inventory; STAI-C: State-trait anxiety inventory for children; WPREMB-R: Weekly parent rating of evening and morning behavior - revised.

## Competing interests

MRK is a member of the Strattera (Atomoxetine) Advisory Board for Eli Lilly Pty. Ltd. The atomoxetine used in the ACTION study was purchased from Eli Lilly Pty. Ltd. MRK has received research support from Brain Resource Company Ltd. for previous ADHD studies. CRC has received consulting fees and stock options in Brain Resource Company Ltd., and is a stock holder in Brain Resource Company Ltd. LMW has received consulting fees and stock options in Brain Resource Company Ltd., and is a stock holder in Brain Resource Company Ltd. She has received Advisory Board fees from Pfizer Inc. SDC has received research support from Brain Resource Company Ltd. for previous ADHD studies. The remaining authors declare they have no competing interests.

## Authors' contributions

All authors contributed to the conceptualization and/or design of the study. TWT drafted the manuscript, while all other authors provided critical revisions and approved the final manuscript.

## References

[B1] Williams LM Hermens DF Thein T Clark CR Cooper NJ Clarke SD Lamb C Gordon E Kohn MR Using brain-based cognitive measures to support clinical decisions in ADHD Pediatr Neurol 2010 42 2 118 26 10.1016/j.pediatrneurol.2009.08.010 20117748

[B2] Rappley MD Clinical practice Attention deficit-hyperactivity disorder N Engl J Med 2005 352 2 165 73 10.1056/NEJMcp032387 15647579

[B3] Svanborg P Thernlund G Gustafsson PA Hagglof B Schacht A Kadesjo B Atomoxetine improves patient and family coping in attention deficit/hyperactivity disorder: a randomized, double-blind, placebo-controlled study in Swedish children and adolescents Eur Child Adolesc Psychiatry 2009 18 12 725 35 10.1007/s00787-009-0031-x 19466476 PMC2770135

[B4] Escobar R Montoya A Polavieja P Cardo E Artigas J Hervas A Fuentes J Evaluation of patients' and parents' quality of life in a randomized placebo-controlled atomoxetine study in attention-deficit/hyperactivity disorder J Child Adolesc Psychopharmacol 2009 19 3 253 63 10.1089/cap.2008.0109 19519260

[B5] National Institute of Mental Health Attention deficit hyperactivity disorder (ADHD) 2008

[B6] Barry RJ Clarke AR Hajos M McCarthy R Selikowitz M Bruggemann JM Acute atomoxetine effects on the EEG of children with attention-deficit/hyperactivity disorder Neuropharmacology 2009 57 7-8 702 7 10.1016/j.neuropharm.2009.08.003 19698723

[B7] Wigal SB Efficacy and safety limitations of attention-deficit hyperactivity disorder pharmacotherapy in children and adults CNS Drugs 2009 23 Suppl 1 21 31 10.2165/00023210-200923000-00004 19621975

[B8] The MTA Cooperative Group A 14-month randomized clinical trial of treatment strategies for attention-deficit/hyperactivity disorder. The MTA Cooperative Group Multimodal Treatment Study of Children with ADHD Arch Gen Psychiatry 1999 56 12 1073 86 10.1001/archpsyc.56.12.1073 10591283

[B9] Geller D Donnelly C Lopez F Rubin R Newcorn J Sutton V Bakken R Paczkowski M Kelsey D Sumner C Atomoxetine treatment for pediatric patients with attention-deficit/hyperactivity disorder with comorbid anxiety disorder J Am Acad Child Adolesc Psychiatry 2007 46 9 1119 27 10.1097/chi.0b013e3180ca8385 17712235

[B10] Hazell P Review of new compounds available in Australia for the treatment of attention-deficit hyperactivity disorder Australas Psychiatry 2004 12 4 369 375 15715810 10.1080/j.1440-1665.2004.02129.x

[B11] Newcorn JH Kratochvil CJ Allen AJ Casat CD Ruff DD Moore RJ Michelson D Atomoxetine/Methylphenidate Comparative Study Group Atomoxetine and osmotically released methylphenidate for the treatment of attention deficit hyperactivity disorder: acute comparison and differential response Am J Psychiatry 2008 165 6 721 730 10.1176/appi.ajp.2007.05091676 18281409

[B12] Hammerness P McCarthy K Mancuso E Gendron C Geller D Atomoxetine for the treatment of attention-deficit/hyperactivity disorder in children and adolescents: a review Neuropsychiatr Dis Treat 2009 5 215 226 19557116 10.2147/ndt.s3896 PMC2695220

[B13] Kratochvil CJ Milton DR Vaughan BS Greenhill LL Acute atomoxetine treatment of younger and older children with ADHD: A meta-analysis of tolerability and efficacy Child Adolesc Psychiatry Ment Health 2008 15 2 25 10.1186/1753-2000-2-25 PMC2556311 18793405

[B14] Garnock-Jones KP Keating GM Spotlight on Atomoxetine in Attention-Deficit Hyperactivity Disorder in Children and Adolescents CNS Drugs 2010 24 1 85 8 10.2165/11203670-000000000-00000 20030421

[B15] Daughton JM Kratochvil CJ Review of ADHD pharmacotherapies: advantages, disadvantages, and clinical pearls J Am Acad Child Adolesc Psychiatry 2009 48 3 240 8 10.1097/CHI.0b013e318197748f 19242289

[B16] Michelson D Allen AJ Busner J Casat CD Dunn D Kratochvil CJ Newcorn J Sallee FR Sangal RB Saylor KE West S Kelsey D Wernicke J Trapp NJ Harder D Once-daily atomoxetine treatment for children and adolescents with attention deficit hyperactivity disorder: a randomized, placebo-controlled study Am J Psychiatry 2002 159 11 1896 901 10.1176/appi.ajp.159.11.1896 12411225

[B17] Montoya A Hervas A Cardo E Artigas J Mardomingo MJ Alda JA Gastaminza X Garcia-Polavieja MJ Gilaberte I Escobar R Evaluation of atomoxetine for first-line treatment of newly diagnosed, treatment-naive children and adolescents with attention deficit/hyperactivity disorder Curr Med Res Opin 2009 25 11 2745 54 19785510 10.1185/03007990903316152

[B18] Wilens TE Newcorn JH Kratochvil CJ Gao H Thomason CK Rogers AK Feldman PD Levine LR Long-term atomoxetine treatment in adolescents with attention-deficit/hyperactivity disorder J Pediatr 2006 149 1 112 9 10.1016/j.jpeds.2006.01.052 16860138

[B19] Newcorn JH Sutton VK Weiss MD Sumner CR Clinical responses to atomoxetine in attention-deficit/hyperactivity disorder: the Integrated Data Exploratory Analysis (IDEA) study J Am Acad Child Adolesc Psychiatry 2009 48 5 511 8 10.1097/CHI.0b013e31819c55b2 19318988

[B20] Ramoz N Boni C Downing AM Close SL Peters SL Prokop AM Allen AJ Hamon M Purper-Ouakil D Gorwood P A haplotype of the norepinephrine transporter (Net) gene Slc6a2 is associated with clinical response to atomoxetine in attention-deficit hyperactivity disorder (ADHD) Neuropsychopharmacology 2009 34 9 2135 42 10.1038/npp.2009.39 19387424

[B21] Svanborg P Thernlund G Gustafsson PA Hagglof B Poole L Kadesjo B Efficacy and safety of atomoxetine as add-on to psychoeducation in the treatment of attention deficit/hyperactivity disorder: a randomized, double-blind, placebo-controlled study in stimulant-naive Swedish children and adolescents Eur Child Adolesc Psychiatry 2009 18 4 240 9 10.1007/s00787-008-0725-5 19156355

[B22] Takahashi M Takita Y Yamazaki K Hayashi T Ichikawa H Kambayashi Y Koeda T Oki J Saito K Takeshita K Allen AJ A randomized, double-blind, placebo-controlled study of atomoxetine in Japanese children and adolescents with attention-deficit/hyperactivity disorder J Child Adolesc Psychopharmacol 2009 19 4 341 50 10.1089/cap.2008.0154 19702486

[B23] Kelsey DK Sumner CR Casat CD Coury DL Quintana H Saylor KE Sutton VK Gonzales J Malcolm SK Schuh KJ Allen AJ Once-daily atomoxetine treatment for children with attention-deficit/hyperactivity disorder, including an assessment of evening and morning behavior: a double-blind, placebo-controlled trial Pediatrics 2004 114 1 e1 8 10.1542/peds.114.1.e1 15231966

[B24] Michelson D Faries D Wernicke J Kelsey D Kendrick K Sallee FR Spencer T The Atomoxetine ADHD Study Group Atomoxetine in the treatment of children and adolescents with attention-deficit/hyperactivity disorder: A randomized, placebo-controlled, dose-reponse study Pediatrics 2001 108 5 e83 10.1542/peds.108.5.e83 11694667

[B25] Spencer T Heiligenstein JH Biederman J Faries DE Kratochvil CJ Conners CK Potter WZ Results from 2 proof-of-concept, placebo-controlled studies of atomoxetine in children with attention-deficit/hyperactivity disorder J Clin Psychiatry 2002 63 12 1140 7 12523874 10.4088/jcp.v63n1209

[B26] Weiss M Tannock R Kratochvil CJ Dunn D Velez-Borras J Thomason CK Tamura R Kelsey D Stevens L Allen AJ A randomized, placebo-controlled study of once-daily atomoxetine in the school setting in children with ADHD J Am Acad Child Adolesc Psychiatry 2005 44 7 647 55 10.1097/01.chi.0000163280.47221.c9 15968233

[B27] Kaplan S Heiligenstein J West S Busner J Harder D Dittmann R Casat C Wernicke JF Efficacy and safety of atomoxetine in childhood attention-deficit/hyperactivity disorder with comorbid oppositional defiant disorder J Atten Disord 2004 8 2 45 52 10.1177/108705470400800202 15801334

[B28] Newcorn JH Spencer TJ Biederman J Milton DR Michelson D Atomoxetine treatment in children and adolescents with attention-deficit/hyperactivity disorder and comorbid oppositional defiant disorder J Am Acad Child Adolesc Psychiatry 2005 44 3 240 8 10.1097/00004583-200503000-00008 15725968

[B29] Ghuman JK Aman MG Ghuman HS Reichenbacher T Gelenberg A Wright R Rice S Fort C Prospective, naturalistic, pilot study of open-label atomoxetine treatment in preschool children with attention-deficit/hyperactivity disorder J Child Adolesc Psychopharmacol 2009 19 2 155 66 10.1089/cap.2008.054 19364293 PMC2857147

[B30] Gualtieri CT Johnson LG Medications do not necessarily normalize cognition in ADHD patients J Atten Disord 2008 11 4 459 469 10.1177/1087054707305314 17934180

[B31] Paul RH Lawrence J Williams LM Richard CC Cooper N Gordon E Preliminary validity of "integneuro": a new computerized battery of neurocognitive tests Int J Neurosci 2005 115 11 1549 67 10.1080/00207450590957890 16223701

[B32] Clark CR Paul RH Williams LM Arns M Fallahpour K Handmer C Gordon E Standardized assessment of cognitive functioning during development and aging using an automated touchscreen battery Arch Clin Neuropsychol 2006 21 5 449 67 10.1016/j.acn.2006.06.005 16904862

[B33] Vaughan B Fegert J Kratochvil CJ Update on atomoxetine in the treatment of attention-deficit/hyperactivity disorder Expert Opin Pharmacother 2009 10 4 669 76 10.1517/14656560902762873 19239401

[B34] Kuczmarski RJ Ogden CL Guo SS Grummer-Strawn LM Flegal KM Mei Z Wei R Curtin LR Roche AF Johnson CL CDC growth charts for the United States: Methods and development National Center for Health Statistics Vital Health Stat 2000 11 246 12043359

[B35] American Psychiatric Association Diagnostic and Statistical Manual of Mental Disorders 1994 Fourth Washington, DC: American Psychiatric Association

[B36] Silverman WK Saavedra LM Pina AA Test-retest reliability of anxiety symptoms and diagnoses with the Anxiety Disorders Interview Schedule for DSM-IV: Child and parent versions J Am Acad Child Adolesc Psychiatry 2001 40 8 937 944 10.1097/00004583-200108000-00016 11501694

[B37] Döpfner M Steinhausen H Coghill D Dalsgaard S Poole L Ralston SJ Rothenberger A ADORE Study Group Cross-cultural reliability and validity of ADHD assessed by the ADHD Rating Scale in a pan-European study Eur Child Adolesc Psychiatry 2006 15 suppl 1 10.1007/s00787-006-1007-8 17177016

[B38] Wood JJ Piacentini JC Bergman RL McCracken J Varrios V Concurrent validity of the Anxiety Disorders Section of the Anxiety Disorders Interview Schedule for DSM-IV: Child and parent versions J Clin Child Psychol 2002 31 3 335 342 10.1207/S15374424JCCP3103_05 12149971

[B39] DuPaul GJ Parent and teacher ratings of ADHD symptoms: psychometric properties in a community-based sample J Clin Child Psychol 1991 20 3 245 253 10.1207/s15374424jccp2003_3

[B40] Conners CK Conners' rating scales - Revised user's manual 1997 North Tonawanda, NY: Multi-Health Systems Inc

[B41] Lovibond SH Lovibond PF Manual for the Depression Anxiety Stress Scales 1995 2 Sydney: Psychology Foundation

[B42] Williams LM Hermens DF Palmer D Kohn M Clarke S Keage H Clark CR Gordon E Misinterpreting emotional expressions in attention-deficit/hyperactivity disorder: evidence for a neural marker and stimulant effects Biol Psychiatry 2008 63 10 917 26 10.1016/j.biopsych.2007.11.022 18272140

[B43] Brain Resource WebNeuro Assessment Manual 2008 1.1 Sydney and San Francisco: Brain Resource

[B44] Spielberger CD Gorsuch RL Lushene RE State-Trait Anxiety Inventory Manual 1970 Palo Alto, CA: Consulting Psychologists Press

[B45] Spielberger CD State-Trait Anxiety Inventory for Children Manual 1973 Palo Alto, CA: Consulting Psychologists Press

[B46] Cross RW Huberty TJ Factor analysis of the State-Trait Anxiety Inventory for Children with a sample of seventh- and eighth-grade students J Psychoeduc Assess 1993 11 3 232 241 10.1177/073428299301100303

[B47] Williams LM Gatt JM Hatch A Palmer DM Nagy M Rennie C Cooper NJ Morris C Grieve S Dobson-Stone C Schofield P Clark CR Gordon E Arns M Paul RH The INTEGRATE model of emotion, thinking and self regulation: an application to the "paradox of aging" J Integr Neurosci 2008 7 3 367 404 10.1142/S0219635208001939 18988298

[B48] Guy W ECDEU Assessment Manual for Psychopharmacology, Revised 1976 Bethesda, MD: U.S. Department of Health, Education and Welfare

[B49] Varni JW Seid M Kurtin PS PedsQL 4.0: reliability and validity of the Pediatric Quality of Life Inventory version 4.0 generic core scales in healthy and patient populations Med Care 2001 39 8 800 12 10.1097/00005650-200108000-00006 11468499

[B50] Wehmeier PM Dittmann RW Schacht A Helsberg K Lehmkuhl G Morning and evening behavior in children and adolescents treated with atomoxetine once daily for attention-deficit/hyperactivity disorder (ADHD): Findings from two 24-week, open-label studies Child Adolesc Psychiatry Ment Health 2009 3 5 10.1186/1753-2000-3-5 19203355 PMC2663547

[B51] Williams LM Tsang TW Clarke S Kohn M An 'Integrative Neuroscience' perspective on ADHD: Linking cognition, emotion, brain and genetics measures with implications for clinical support Expert Rev Neurother 2010 10 10 1607 1621 10.1586/ern.10.140 20925475

[B52] Chang K Nayar D Howe M Rana M Atomoxetine as an adjunct therapy in the treatment of co-morbid attention-deficit/hyperactivity disorder in children and adolescents with bipolar I or II disorder J Child Adolesc Psychopharmacol 2009 19 5 547 51 10.1089/cap.2009.0030 19877979

[B53] Faraone SV Biederman J Spencer T Michelson D Adler LA Reimherr F Seidman L Atomoxetine and Stroop task performance in adult attention-deficit/hyperactivity disorder J Child Adolesc Psychopharmacol 2005 15 4 664 670 10.1089/cap.2005.15.664 16190797

[B54] Spencer T Biederman J Wilens T Prince J Hatch M Jones J Harding M Faraone SV Seidman L Effectiveness and tolerability of tomoxetine in adults with attention deficit hyperactivity disorder Am J Psychiatry 1998 155 693 695 9585725 10.1176/ajp.155.5.693

[B55] Arnold LE Aman MG Cook AM Witwer AN Hall KL Thompson S Ramadan Y Atmoxetine for hyperactivity in autism spectrum disorders: placebo-controlled crossover trial J Am Acad Child Adolesc Psychiatry 2006 45 10 1196 205 10.1097/01.chi.0000231976.28719.2a 17003665

